# Factors influencing postnatal Option B+ participation and breastfeeding duration among HIV-positive women in Lilongwe District, Malawi: A qualitative study

**DOI:** 10.1371/journal.pone.0175590

**Published:** 2017-04-14

**Authors:** Valerie L. Flax, Gloria Hamela, Innocent Mofolo, Mina C. Hosseinipour, Irving F. Hoffman, Suzanne Maman

**Affiliations:** 1 Department of Nutrition, Gillings School of Global Public Health, University of North Carolina, Chapel Hill, North Carolina, United States of America; 2 RTI International, Research Triangle Park, North Carolina, United States of America; 3 UNC Project – Malawi, Lilongwe, Malawi; 4 Department of Medicine, School of Medicine, University of North Carolina, Chapel Hill, North Carolina, United States of America; 5 Department of Health Behavior, Gillings School of Global Public Health, University of North Carolina, Chapel Hill, North Carolina, United States of America; University of Cincinnati College of Medicine, UNITED STATES

## Abstract

To ensure the health of mothers and children, prevention of mother-to-child HIV transmission (PMTCT) programs test women for HIV, engage HIV-positive women in care, and promote recommended breastfeeding practices. Under Malawi’s Option B+ PMTCT program, ~20% of women are lost-to-follow-up (LTFU) and little is known about their breastfeeding practices. The purpose of this study is to describe facilitators and barriers to Option B+ participation and how participation influences breastfeeding duration. We conducted in-depth interviews with HIV-positive women in Option B+ (n = 32) or LTFU from Option B+ (n = 32). They were recruited from four government clinics in Lilongwe District and had a child aged 0–23 months. Women in Option B+ had better disclosure experiences and more social support than LTFU women. The most common reasons for LTFU were fear of HIV disclosure, anticipated or experienced stigma, and insufficient social support. Other reasons included: non-acceptance of HIV status, antiretroviral therapy (ART) side effects, lack of funds for transport, and negative experiences with clinic staff. Worries about possible transmission, even while on ART, influenced timing of weaning for some women in Option B+. Despite their knowledge of the risk of HIV transmission to the child, most LTFU women continued to breastfeed after stopping ART because they considered breastmilk to be an important source of nutrients for the child. Given that HIV-positive Malawian women LTFU from Option B+ breastfeed in the absence of ART, efforts are needed to use evidence-based strategies to address the barriers to Option B+ participation and avert preventable transmission through breastmilk.

## Introduction

Antiretroviral therapy (ART) and optimal infant feeding practices are the two main strategies recommended by the World Health Organization (WHO) for limiting HIV transmission from mother to child and improving the HIV-free survival of exposed infants and young children [1,2]. Prevention of mother-to-child transmission (PMTCT) programs in low- and middle-income countries with high HIV prevalence typically implement these guidelines through same-day HIV testing and treatment initiation for pregnant or breastfeeding women and through group or individual infant feeding counseling. Option B+ is currently the most widely used PMTCT strategy [3]. It offers lifelong ART regardless of women’s immunologic or clinical disease stage [2]. At the time of this study, WHO recommended that HIV-positive women should exclusively breastfeed their infants until six months and continue breastfeeding until 12 months [1]. Malawi modified the guidelines to extend continued breastfeeding to 24 months [4]; the same extended period of breastfeeding was recently adopted under the updated WHO HIV and infant feeding guidelines [5].

Option B+ has increased PMTCT participation and ART coverage among pregnant HIV-positive women in Malawi [6–8]. However, approximately 20% of Malawian women who are tested and enrolled in Option B+ are lost to follow-up (LTFU), with more than half of these stopping ART [9]. Loss to follow-up occurs most frequently during the first three months after women start the program and among women with high CD4 counts or those who enter PMTCT and initiate ART while breastfeeding [10,11]. Studies of barriers to PMTCT participation found that some of the common reasons women dropped out were fears of involuntary HIV disclosure; stigma from family and community members; negative interactions with health staff; lack of support from husbands; and difficulties accessing services [11–17].

While these studies provide useful information for improving PMTCT programs, there are some important gaps. Few studies of challenges to PMTCT participation were conducted within the context of Option B+ [9,18–20] and only two of these collected qualitative data on the reasons women drop out of the program [18,20]. There are only a few studies that focused on challenges to PMTCT participation up to 18–24 months postpartum [17,21]. In addition, little is known about how ART use or the decision to stop using ART influences breastfeeding practices or, conversely, whether the decision or perceived need to breastfeed contributes to ART use. The aims of this study were to identify facilitators and barriers to Option B+ participation and to document the duration of breastfeeding among HIV-positive women with children 0–23 months of age who were participating in or had been LTFU from Option B+.

## Methods

### Study setting and sample

We conducted qualitative in-depth interviews with 64 HIV-positive women from July 2014-January 2015. Half of them were participating in Option B+ and the other half were LTFU from the Option B+ program. Women were enrolled in the study if they were ≥ 18 years of age, lived in the catchment areas of four government clinics (two urban, two rural) in Lilongwe District, Malawi, and had a child 0–23 months of age. Women who were LTFU were recruited if they conformed to the Malawi Ministry of Health’s definition (i.e., failure to attend the clinic for >60 days since their last scheduled PMTCT visit). We purposively sampled equal numbers of women with children in four age groups (0–5, 6–11, 12–17, and 18–23 months) to ensure that we collected information about feeding practices throughout the period when women are usually enrolled in PMTCT in Malawi. We planned our sample sizes in advance so that we would have eight women with a child in each age group among women in PMTCT and among LTFU women. Our sub-group sample sizes exceeded the minimum of six interviews needed to achieve saturation [22], and we found during the analysis that we had attained saturation.

Research assistants responsible for data collection were Malawian university graduates trained on qualitative interviewing techniques and the specifications of this study for five days. They recruited PMTCT participants as they arrived at the clinic for their regular PMTCT visits and checked the women for eligibility. Research assistants approached and recruited women until they reached the target enrollment (e.g., two women per child age group per clinic). Different strategies were employed in the four clinics to recruit LTFU women for the study. Health workers used patient lists and contacted LTFU women starting from those who were most recently lost from the program. In the largest, urban clinic, health care workers contacted LTFU women by phone to schedule interviews. In the other clinics, cell phone numbers were used when available, but most often a health care worker or health surveillance assistant went to the woman’s home to schedule the interview appointment. Interviews with women in PMTCT took place at the clinics, whereas interviews with LTFU women took place either in the women’s homes or at a clinic.

### Ethical issues

For interviews with PMTCT participants and LTFU women at clinics, research assistants conducted them in a private room or an area away from other patients. For interviews at LTFU women’s homes, research assistants used cover stories so that they did not reveal the topic of the interview if family or community members asked questions about their presence. They stopped the interview temporarily if anyone entered the room while the interview was underway.

Research assistants obtained written or thumb-printed informed consent from all participants after reading the Chichewa consent aloud and answering participants’ questions. The Institutional Review Board at the University of North Carolina (#13–3615) and the Malawi Ministry of Health’s National Health Sciences Research Committee (NHSRC #1231) provided ethical approval for the study.

### Data collection

The in-depth interviews were conducted by four Malawian research assistants. Interview guides for PMTCT participants and LTFU women included open-ended questions followed by probes to help interviewers collect more detailed information on women’s experiences with PMTCT services (facilitators and barriers), HIV disclosure experiences, support for PMTCT participation from family or friends, breastfeeding practices, and use of ART. For LTFU women, the guides also included questions on reasons for and timing of stopping PMTCT participation. The guides were prepared in English and translated into Chichewa. They were piloted and the translations were adjusted to improve clarity before beginning data collection. Interviews were digitally recorded and lasted 20–45 minutes. They were transcribed verbatim in Chichewa then translated into English.

### Data analysis

We entered in-depth interview transcripts into Dedoose (Version 5.0.11, SocioCultural Consultants LLC) and analyzed them using thematic analysis [23]. The coding team included the first author and three students at the University of North Carolina who were trained in qualitative analysis techniques for this project. The coding team met weekly to discuss the codes and make changes to the codebook to capture new ideas that emerged from the data. We initially used deductive codes based on the question guides and then conducted a second round of coding using inductive codes that emerged from the data during the deductive coding process. To ensure consistency across coders, the first author independently coded ten percent of the interviews to compare with students’ coding. Discrepancies were discussed by the team and a consensus was reached on the final code definitions and application. We entered coded data into data matrices to facilitate identification of themes, comparisons across groups, and selection of illustrative quotes [24].

### Trustworthiness of the findings

We took several steps to ensure the trustworthiness of our findings [25]. The research team was comprised of Malawians and Americans who have long-term research experience in Malawi. The study from which the data were drawn included observations and interviews with health workers, which could be used to triangulate the findings [26]. We selected different types of clinics and use purposive sampling to improve transferability of the results to other settings [27]. We improved the dependability of repeating the findings by checking the consistency of our coding and making sure we achieved data saturation. Further, our findings align with other research in Malawi [12,13,18,21] and have been corroborated by subsequent research [28].

## Results

### Participant characteristics and breastfeeding practices

The characteristics of enrolled PMTCT participants and LTFU women were generally similar. On average women in the study were 27 years of age, had six years of education, had given birth to three children, and owned two household items out of a list of 10 (including electricity, paraffin lamp, radio, television, phone, mattress, table and chairs, sofa, and refrigerator). About one-fifth of PMTCT participants and LTFU women had been diagnosed with HIV > 2 years ago, either during a previous pregnancy or as a result of illness. Slightly more LTFU women than participants were not married and reported household food insecurity, which was present among >80% of the women in both groups.

Among LTFU women, three participants did not admit they had stopped participating, two never started taking ART, six stopped taking ART during pregnancy, and one stopped at the birth of the child. Other LTFU women stopped taking ART when the child was 0–5 months (n = 10), 6–11 months (n = 7), or ≥ 12 months (n = 3).

Patterns of breastfeeding duration were similar among PMTCT participants and LTFU women. Of the 32 PMTCT participants in our sample, 27 were still breastfeeding at the time of the interview. Five PMTCT participants decided to wean early, and four of the participants who were still breastfeeding planned to wean the child before 24 months. Those participants who weaned early or planned to wean early had a variety of reasons, but the most common was that they were worried about HIV transmission even though they were on ART. For example, PMTCT participant #28 said, *“I thought of stopping to breastfeed my child [at 9 months] because I can suddenly transmit the virus to him*.*”* Out of 32 LTFU woman, 24 were still breastfeeding at the time of the interview, although they were no longer taking ART. Eight LTFU woman weaned the child early and half of these had either ceased breastfeeding prior to or concurrently with stopping use of ART. The number of months LTFU women breastfed without ART coverage ranged from 0 to 11 and averaged four months at the time of the interviews.

### Facilitators and barriers to Option B+ participation

Key facilitators to Option B+ participation were knowledge of the health benefits of ART, good HIV disclosure experiences, support from family members, and good experiences with health workers. Fear of HIV transmission through breastmilk did not motivate LTFU women to return to care. The most common barriers to participating in Option B+ were fear of HIV disclosure, fear of HIV stigma, lack of support from family, and issues related to travel and accessing ART. Other barriers included: lack of self-acceptance of HIV status, ART side effects, and negative experiences with health workers. Based on these themes, we grouped facilitators and barriers to Option B+ participation into three categories: individual, family, and health facility.

#### Individual

The most frequent individual issues related to Option B+ participation were knowledge of ART benefits, breastfeeding decision-making, lack of self-acceptance of HIV status, and ART side effects. For women in PMTCT, individual women’s knowledge of the benefits of taking ART for their own health and for preventing transmission to their children through breastmilk was an important facilitator of PMTCT participation. For example, one PMTCT participant (#27) explained, *“I feel good because I protect my baby from getting the virus*. *If I don’t take the drugs and breastfeed my child*, *it is obvious the virus will be transmitted*.*”* Most PMTCT participants felt comfortable with breastfeeding while on ART and wanted to continue breastfeeding until 24 months. PMTCT participant #26 stated, *“I will wean at 24 months because the baby will be grown and will have some knowledge about eating food*. *Also*, *we were told here [at the clinic] that we can breastfeed up to 24 months*.*”*

LTFU women who continued breastfeeding knew that they could transmit HIV to the infant through breast milk and many of them were worried about this. For example, LTFU woman #59 explained, *“When I stopped taking the drugs*, *I was not okay with it*. *I had worries that as I am continuing to breastfeed my child I will transmit the virus to him*.*”* Despite their worries, fear of HIV transmission through breastmilk did not prompt LTFU women to return to care and many of those who were still breastfeeding planned to continue until the child was 24 months of age. They explained that they did this because the children were still young and needed breastmilk to be healthy. LTFU woman #33 described her reasoning for continued breastfeeding in the absence of ART, *“I was breastfeeding him without any protection*. *But because of the kind of food [our family has available]*, *it was difficult for me to wean him as I thought that the child might become unhealthy*.*”*

Other individual issues that were barriers to PMTCT participation for some LTFU women were unwillingness to start taking ART because the woman had not accepted her HIV status and ART side effects. Each of these issues were mentioned by a few of the LTFU women in our sample. The PMTCT strategy to test pregnant and breastfeeding women for HIV and start them on ART immediately made some women feel like they did not have time to adjust to the information about their status. For example, LTFU woman #62 explained, *“Yes*, *they gave me [ART drugs]*, *but at that time I was not ready to start taking them*, *so I just kept them*.*”* Many women in our sample said they experienced ART side effects, especially when they first started taking the drugs. One PMTCT participant (#1) described her experiences with ART, *“My head used to feel dizzy at first when I took [the drugs]*, *but now it’s all fine…It just happened that everything started to be okay from nowhere*, *but during the first days I was not okay*.*”* For a few LTFU women, the side effects were prolonged or so severe that they decided to stop taking ART. LTFU woman #52 had persistent side effects. She explained, *“I used to take the drugs and when I took them I felt dizzy*. *I could not work properly*, *so I stopped taking them*.*”*

#### Family

Family and social issues that facilitated or impeded PMTCT participation centered on the interrelated issues of HIV disclosure, HIV stigma, social support, and marital relationships. All PMTCT participants had disclosed their HIV status, whereas four LTFU women had not disclosed to anyone. More PMTCT participants (n = 27) than LTFU women (n = 19) had disclosed their HIV status to their husbands, and more of them also disclosed to their mothers, siblings, other relatives, and friends or neighbors. PMTCT participants had fewer negative disclosure experiences than LTFU women. Twenty husbands of PMTCT participants reacted positively when their wives disclosed their HIV status, two were neutral, and three reacted negatively.

In contrast, LTFU women reported that 11 husbands accepted the news of their wife’s HIV status, none were neutral, and nine reacted negatively.

Husbands’ and other relatives’ initial reactions and subsequent actions in relation to women’s HIV status seemed to play an important role in terms of social support for women to participate in PMTCT. Many PMTCT participants and some LTFU women received positive responses when they disclosed to family members. For example, a PMTCT participant explained,

“*They [my husband and sister] were fine with it and they have been encouraging me since [I disclosed]…They encourage me to accept [my HIV status] as that is the way things are and [I should] never be worried. So, I find encouragement from these people*.”

PMTCT participant #6

Husbands and other family members often supported women’s participation in PMTCT by reminding them to take their ART or to go to the clinic to collect the drugs. One woman explained that her husband helps her as follows:

“*He encourages and asks me at bedtime if I have taken the drugs*, *since we are given a specific time to take the drugs*. *If I haven’t*, *he goes and gets the drug and water and gives it to me*.*”*

PMTCT participant #15

Husbands of women in PMTCT may have been more supportive than those of LTFU women because all HIV-positive husbands of PMTCT women were taking ART (13/13), whereas less than half of HIV-positive husbands of LTFU women were on treatment (5/12). When both members of the couple were HIV-positive and on treatment, they could support each other to take their ART regularly.

When PMTCT participants did not receive encouragement from their husbands or if they did not currently have a partner, they were encouraged or supported by other relatives or friends. A married PMTCT participant (#14) explained, *“When I disclosed to my husband that I am HIV positive*, *he ran away*. *I disclosed to my grandparents*, *who said I should move and stay with them*.*”* Another PMTCT participant who was widowed said,

“*At first I disclosed at home to my elder sister then I managed to disclose to my friends, the ones I chat with… These people they just encouraged me that I should continue taking the drugs the way they have been prescribed at the hospital*.”

PMTCT participant #26

In contrast, many of the LTFU women were not in supportive environments and their husbands’ reactions made it difficult for them to stay on treatment. Their husbands tended to blame them for contracting the virus and stigmatized them. The following quote illustrates the type of negative response and stigmatizing reaction from some husbands when their wives disclosed their HIV status:

“*When I came back from the hospital, my husband asked me how I was. I told him that I was diagnosed with the virus and then he changed. He started stigmatizing me… He started going to bars and he would come home in the morning after spending the night there and sometimes he would not come home. In some cases, he would not be at home for a week or a month… He did not receive the news well and was very angry with me*.”

LTFU woman #48

Some LTFU women were so worried about their husbands’, relatives’, or friends’ responses to their HIV status that they either delayed disclosing or did not disclose at all. LTFU woman #33 was worried about the consequences of disclosure on her marriage, but had an unexpectedly positive response when her husband discovered her ART.

“*It was really a problem, because when I was given the results of my blood test here at the hospital it was very difficult for me to tell my husband, since he was not an understanding man. So, I kept quiet for months without explaining anything to him… By grace, he found the drugs [ART]. That was when he asked me as to what I was doing with the drugs and I told him that I had received the drugs from the hospital…as I have been found with the virus. Then he said, ‘Why were you not telling me? I am ready to accept anything if things are like this. I might be on the side of going for testing, too, so you were wrong [to hide]. You could have just explained.’ And I saw that as a response from God since the thing that was causing worry to me was what would happen if he finds out. Maybe my marriage would end*.”

LTFU woman #33

Some women who did not disclose to their husbands were justified in their fear of disclosure. For LTFU woman #42, her husband’s discovery of her drugs resulted in physical violence. She explained, *“My husband found the drugs and he asked me about them*. *I tried to explain*, *but we ended up fighting*. *Look around my neck [showing interviewer scars on her neck]*.*”* She ultimately left the PMTCT program because of the fights she had with her husband.

Non-disclosure was often related to fear of HIV stigma and made it difficult for women to collect their ART from the clinic or hide taking ART. The following quotes are typical of the interconnectedness of HIV stigma and disclosure and how this impacted LTFU women’s ART use:

“*My husband and the people from the community where I live, if they see me going to get ART, since I haven’t revealed [my HIV status] to them, they will discriminate against me and say bad things about me*.”

LTFU woman #36

“*I was away from home. I went to my mother-in-law’s place, so I thought it was not wise for me to get my drugs [from the health center] because I did not disclose my HIV status to her*.”

LTFU woman #35

Several LTFU women described unstable marital relationships that influenced their PMTCT participation. The following two women explained how they started taking ART, but then stopped due to changes in their relationship with their husbands.

“*I was taking [ART] before, but then I had an argument with my husband, so I went to my home village and I stopped. It’s been four months since I took them*.”

LTFU woman #37

“*I came to the hospital for HIV testing and I was found positive, so I started receiving ART. I received the drugs for only one month. Then my husband left me and married another woman, so I stopped taking the drugs and coming here to collect the drugs all together*.”

LTFU woman #44

#### Health facility

The main health facility facilitators and barriers to PMTCT participation were the women’s interactions with health workers and the amount of time they spent at the clinic. Most respondents–both women in PMTCT and LTFU women–had positive or neutral experiences with the health workers who provide PMTCT services in the study clinics. For example, one respondent stated,

“*They [the health workers] were treating us so nicely by greeting us, giving us the drugs, and encouraging us that taking ART is not the end of our lives, but the beginning of another chapter in life*.”

LTFU woman #42

Among PMTCT participants, none had negative experiences with health workers, but two of them were worried about the length of time they spent at the clinic during PMTCT visits. One explained what happens at the clinic,

“*When we get here the health workers are very slow in delivering their services. They don't respect the fact that we have small children. They don't pay attention to us until lunch time and then they leave for lunch without helping us*.”

PMTCT participant #9

One-quarter of the LTFU women described negative interactions with health workers. One woman stopped participating in PMTCT because a health worker disclosed her HIV status in front of other patients. She explained,

“*The health workers did not speak well to me. They asked me in the presence of other women at the under-five clinic, ‘Have you collected your ART?’ This made me mad as I felt like I was being embarrassed in front of other people*.”

LTFU woman #36

For a few other women, a negative or harsh tone by health workers in the PMTCT program, especially if they missed a clinic visit, was a barrier to participation. For example, LTFU woman #33 stated, *“I skipped a certain visit [to the hospital]*, *as I had gone somewhere*. *When I came back*, *I met the doctor and the way he spoke to me*, *I left again… I decided to just quit*.*”* Several LTFU women in our sample talked about their fear of being scolded by health workers if they went back to the clinic to restart PMTCT.

Travel and transport were the most frequent access issues related to PMTCT participation. Many LTFU women in our sample stopped taking ART because they moved or traveled to visit or help relatives in another part of Malawi or in Mozambique. Some could not access ART at a clinic near where they were visiting because they did not have a transfer letter and others (as noted in one of the quotes above) were afraid to access ART when staying with people who did not know their HIV status. One woman explained,

“*I went to live with my mother. Unfortunately, I finished the drugs I received, but I wasn’t allowed to receive drugs from a different hospital because they were demanding a referral letter and health passport book, which I forgot*.”

LTFU woman #56

Another woman (LTFU #49) stated, *“I took [ART] for six years*. *I stopped because I went to Mozambique for some weeks*. *The drugs I carried got finished*.*”*

For a few PMTCT participants and LTFU women finding money for transport to the clinic to attend PMTCT visits was problematic. Five LTFU women cited transport as one of the reasons they stopped participating in Option B+. One of them explained,

“*[My husband] said it was a good thing that I know my HIV status. He was not unhappy, because if he was, then I would not be coming to the hospital. But I stopped because I had transport problems*.”

LTFU woman #57

A few LTFU women who had transport issues also faced multiple problems simultaneously and simply could not overcome them in order to stay in the PMTCT program. One of these women described her situation as follows:

“*I was in deep trouble. I had a job and I was laid off. Also, my child got sick while that was happening and I was evicted from my house. I didn’t have the transport to travel from here to [the clinic] and from [the clinic] to my house*.”

LTFU woman #63

Women such as this one lack a social safety net or support from their family to see them through a difficult time and facilitate continued PMTCT participation.

## Discussion

The facilitators and barriers to PMTCT participation described in this study are similar to those reported in other studies of PMTCT in sub-Saharan Africa in settings with Option B+ and under other PMTCT options [9,11–20,29–33]. Our findings are consistent with studies reporting that knowledge of the health benefits of ART is an important facilitator of women’s PMTCT participation [18,29–31]. Like other research, this study found that fear of HIV disclosure, fear of HIV stigma, and lack of social support were some of the most common barriers to PMTCT participation [12–14,16,18–20,29,31,32]. Although cost of transport to the clinic, ART side effects, and negative treatment by health workers were also mentioned as barriers, each of these issues impacted only a few women and they were not the main barrier for most LTFU women in our sample, as opposed to other studies [9,12,16,18–20,29,32–34].

This study adds to the literature by comparing experiences of PMTCT participants and LTFU women. Through these comparisons, we found that positive disclosure experiences and support from family members, whether from husbands or others, are key to helping women overcome barriers to PMTCT participation. Women who had not disclosed their HIV status to members of their household had trouble hiding daily use of ART because they feared inadvertent disclosure and the resulting stigma and other negative consequences, such as divorce, which is commonly associated with PMTCT in some parts of Malawi [35]. We also showed that disclosure, stigma, and lack of social support remain barriers to Option B+ participation at all stages of the postpartum period, up to 24 months. These barriers are interrelated and woven into some of the other reasons for stopping PMTCT participation. For example, when women’s HIV status was disclosed by health staff in a public space during a health facility visit, the issues of disclosure, stigma, and negative interactions with health workers intersected to create an overwhelming barrier to Option B+ participation. Women who stayed in the PMTCT program also had fears about disclosure and stigma, but they often overcame them, in part, through social support. Our findings point to the importance of providing or bolstering social support for HIV-positive women with young children. Programs offering social support can improve women’s motivation, facilitate disclosure, and reduce stigma, all of which may contribute to their PMTCT participation [36].

The other important contribution of this study was the comparisons we made in breastfeeding duration between women in the Option B+ program and those who were LTFU. Women in our sample continued to breastfeed regardless of whether they were participating in PMTCT. Most of the LTFU women, who never started taking ART or who stopped using it, realized that they were putting their child at risk of HIV transmission. However, they did not feel like they had a choice because the majority of them lived in households with regular food insecurity and they did not have the ability to offer the child infant formula or sufficient other foods. While food insecurity plays an important role, women’s decisions about breastfeeding and weaning are also influenced by cultural norms. Early weaning is not appropriate in Malawian culture and breastfeeding is an important part of being a mother [21,37]. Women who wean early are assumed to have become pregnant again with a short birth interval or to be HIV-positive, both of which have negative social consequences [38,39]. In this context, breastfeeding norms and lack of foods that can adequately replace breastmilk override worries about the child getting HIV through breastmilk. Programs could emphasize the importance of women staying on ART to protect the child from transmission through breastmilk, but that information is not sufficient without addressing the underlying issues of fear of HIV disclosure, experiences of HIV stigma, and other barriers to participation.

This study had some limitations. First, one of our aims was to study duration of breastfeeding among women in PMTCT and those who were LTFU. We purposely sampled women with children in different age groups within the range from 0–23 months. This made our PMTCT and LTFU groups comparable, but also limited our ability to document the exact timing of weaning, since most of the women had not yet weaned their child and many planned to continue breastfeeding until 24 months. Second, we faced challenges with recruiting LTFU women, especially in the urban clinics. The LTFU women we interviewed were open about sharing their reasons for stopping participation, but perhaps women facing even more serious issues, like intimate partner violence, were not included.

## Conclusions

The findings of this study indicate the importance of hearing the perspectives of both PMTCT participants and LTFU women to better understand facilitators and barriers to participation in Option B+. Achieving higher retention rates in Option B+ will likely require efforts to scale up evidence-based interventions that address the underlying individual, family, and facility issues that influence participation. Efforts to increase continued PMTCT participation should include strategies to reduce HIV stigma in health facilities and communities [40–42] and to facilitate HIV disclosure through couple counseling and tracing and testing programs [43,44]. In-service training for health workers on maintaining confidentiality and on interacting with patients in a positive way is needed [34]. Mechanisms for promoting social support to HIV-positive women through their families or by building alternate supporting mechanisms (e.g., through HIV-positive mentor mothers) can facilitate PMTCT participation [45]. Using single or combined intervention strategies to encourage Malawian women to stay in PMTCT may contribute to the reduction of HIV transmission, because LTFU women are likely to continue breastfeeding even when no longer on ART.
